# Homœopathic Medical College of Missouri

**Published:** 1894-12

**Authors:** 


### HOMŒOPATHIC MEDICAL COLLEGE OF MISSOURI.

The opening exercises of the Homœopathic Medical College of Missouri took place at the school building on Jefferson Avenue and Howard Street, St. Louis, Mo. The attendance was the largest in the history of the College, which was founded in 1857. Dr. Wm. C. Richardson, the Dean, and the other members of the Faculty were present.

Dr. W. B. Morgan, Professor of Surgery, delivered the address to the students. After remarking upon the various causes that incite young men to select the medical profession as their life work, Dr. Morgan said:

"From your text-books and teachers you will gradually learn the details of our art. A deluge of facts and opinions will be put before you that it will take years for you to digest and fully understand. Accept the facts and treasure the opinions of your teachers, but do not allow your minds to become biased concerning any of the medical theories. Many old-school doctors, who do not know anything about it, are as afraid of Homœopathy as a mad dog is of water, and some homœopathic doctors are just as rabid. Now, I hope you will never allow yourselves to get into such a state of mind that you cannot and will not be able to weigh fairly the evidence on both sides of any question. Such an ability is a requisite to true student life. Without it learning may be a fabric of delusions. The history of medicine in the past has been a succession of fanciful theories. There is no dearth of theories at the present time, nor of bigotry concerning them, but there is a new spirit of judicial investigation growing in the profession. By the microscope and other searching means of investigation the theories concerning disease and its treatment are being put to a test that will forever dispel many of the delusions and establish many facts in medicine. Most of us in the homœopathic school have by our practice and teaching admitted that Hahnemann, like most enthusiasts, claimed too wide a scope for the homœopathic law. Most of his followers employ many resources not in keeping with the law, but a hundred years' experience has proved this, that all these other resources are empirical, and that there is no law in therapeutics but that of similars. The law is a trade-mark that we are proud of. Scientific investigation may help to define its province, but cannot overthrow it. Old-school prejudice may keep up partisan feeling for awhile longer, but it cannot suppress the truth. Already ptomaines are recognized old-school remedies for the diseases in which they are produced, an ever-increasing amount of their therapeutics is adopted from our text-books, and there are few allopathic doctors who do not do considerable homœopathic prescribing, though some of them do not know it, and some of them would not own it if they did. The general recognition and acceptance of whatever truth there is in Homœopathy and the giving of due credit to those who have established that truth is not far distant. The elimination of bigotry from our ranks will do much to hasten that end, and I urge you, who are just coming to us, to carefully avoid any such tendency."

The Doctor concluded his address with a few words of advice of a general nature.

